# Propofol maintains Th17/Treg cell balance in elderly patients undergoing lung cancer surgery through GABAA receptor

**DOI:** 10.1186/s12865-022-00490-8

**Published:** 2022-11-25

**Authors:** Can Cui, Dengwen Zhang, Ke Sun, Yi Zhu, Jindong Xu, Yin Kang, Guangyan Zhang, Yujin Cai, Songsong Mao, Ruichun Long, Jue Ma, Song Dong, Yi Sun

**Affiliations:** 1 Department of Anesthesiology, Guangdong Provincial People’s Hospital, Guangdong Academy of Medical Sciences, 96 DongChuan Road, Guangzhou, 510080 China; 2 Guangdong Lung Cancer Institute, Guangdong Provincial People’s Hospital, Guangdong Academy of Medical Sciences, 96 DongChuan Road, Guangzhou, 510080 China

**Keywords:** Lung cancer, Propofol, Elderly, Th17/Treg, γ-aminobutyric acid A receptor, Metastasis, Invasion, Migration

## Abstract

Propofol is widely used in clinical anesthesia due to its advantages of rapid onset and less adverse reactions. This study focused on the role of propofol in the balance of Th17/Treg in elderly patients with lung cancer during perioperative period. Patients undergoing lung cancer surgery were anesthetized by propofol or sevoflurane. Veinal blood was collected at different time points to evaluate the changes of Th17/Treg cell. Propofol better maintained the balance of Th17/Treg in vivo. The peripheral blood of patients with lung cancer was collected in vitro before surgery. Cluster of differentiation (CD)4^+^ T cells were obtained and then treated with propofol at different concentrations and γ-aminobutyric acid A (GABAA) receptor antagonists. Propofol affected Th17/Treg cell balance by increasing Th17 cells, decreasing Treg cells, thus elevating Th17/Treg ratio, and inhibited invasion and migration of lung cancer cells through GABAA receptor, which was counteracted by GABAA receptor inhibitors. Subsequently, tumor in situ model of lung cancer in aged mice was established. Propofol anesthetized mice had lower change of Th17/Treg ratio, higher survival rate and less metastasis. In brief, propofol regulated balance of Th17/Treg in elderly patients undergoing lung cancer surgery through GABAA receptor. Additionally, propofol could inhibit metastasis of lung cancer.

**Supplementary Information:**

The online version contains supplementary material available at 10.1186/s12865-022-00490-8.

## Introduction

Lung cancer is the most frequent malignancy worldwide, representing the primary cause of cancer-related mortality [1]. At present, surgical resection is a crucial therapy for patients with lung cancer, but recurrence and metastasis after surgery remain the leading obstacles to improve survival rate [2], the reason of which is that surgical trauma can interfere with immune functions, inhibit postoperative adaptive immune response and anti-tumor immunity. Besides, the imbalance of CD4^+^ T cell subsets plays a major role in adaptive immune dysfunction [3].

Cluster of differentiation (CD)4^+^ T cells are key regulators of the adaptive immune system, which play different roles in modulating the reactions of microorganisms [4]. CD4^+^ T cells differentiate into 4 subsets: Th1, Th2, Th17 and Treg cells, involving in various types of immune responses [5]. There is ample evidence that the accumulation of immunosuppressive lymphocytes, represented by regulatory T cells (Treg), which participate in maintaining the immunological self-tolerance and immune homeostasis, is related to advanced tumor growth and poor prognosis in several kinds of malignant tumors, including lung cancer [6–8]. Loss of intratumoral Tregs increases antitumor immunity and tumor rejection in mouse models [9]. Similar to Tregs, T helper IL-17-producing cells (Th17) are engaged in tumor immunology, and might promote inflammation in a series of autoimmune diseases. Recently, Th17 cells and their hallmark cytokine have been reported to be implicated in both pro- and anti-tumorigenic processes [10]. Th17 might produce an indirect immune effect in anti-tumor immune responses [11]. Th2 lymphocyte expression increases in trauma and surgery-associated physical injury, leading to impairment of cell-mediated immunity (CMI), and increase of Treg cell expression plays a fundamental role in mediating the immunosuppression [12]. However, the tendency of Th17/Treg ratio in lung cancer and complete resection of lung cancer remains unclear [13–15]. The characteristics and mechanisms of Th17/Treg imbalance may vary in different pathologic types of lung cancer and different tissues (such as tumor tissues and peripheral blood). Therefore, exploring the changes of Th17/Treg ratio afer surgical trauma is of great significance, which helps find effective therapeutic strategy to improve immunosuppression after surgical trauma.

Emerging evidence has shown that different anesthesia methods affect perioperative immune function, tumor cell proliferation, invasion and metastasis, thereby affecting the long-term prognosis of patients [16]. Especially for the cancer patients, the immunosuppression caused by anesthetics can promote the metastasis of residual malignant cells, thus deteriorating the prognosis [17]. Elderly patients are more vulnerable to negative effects of surgery and anesthesia because their physiological elasticity is lower and the incidence rate of combined diseases is higher [18]. Moreover, the cancer cells of the elderly patients present a more aggressive phenotype and their surrounding microenvironment is in a higher immunosuppressive state [19]. Therefore, the anesthesia should be determined prudently for the surgery of elderly patients with lung cancer.

Propofol is an effective intravenous anesthetic, widely utilized in anesthesia induction, maintenance and sedation [20]. In recent years, increasing attention has been paid to the role and mechanism of propofol in anti-tumor progress in vitro and in vivo [21]. Abundant researches show that compared with inhalation anesthesia, propofol total intravenous anesthesia does not suppress the immune system, which can reduce the risk of cancer recurrence [22–24].

Recently, exploring the influence of anesthesia on the balance of cytokines in tumor patients during perioperative period is becoming the hot issue of anesthesiology research. However, relatively little is known about the function of propofol on Th17/Treg cell balance in elderly patients with lung cancer. This study herein investigates the effects of sevoflurane inhalation anesthesia and propofol total intravenous anesthesia on the Th17/Treg cell balance in patients with lung cancer during perioperative period, which shall shed light on the rational use of anesthesia in clinical practice.

## Materials and methods

### Ethics statement

The study was approved by the Ethics Committee of Guangdong Provincial People’s Hospital (No: GDREC2012116H(R1)) and was conducted according to the Declaration of Helsinki. Informed consent was signed by all patients. All animal experiments are approved by the Ethics Committee of Guangdong Provincial People’s Hospital. The study was carried out in compliance with the ARRIVE guidelines. All procedures performed on animals were carried out in accordance with relevant guidelines and regulations.

### Participants and anesthesia surgery management

Altogether 36 patients with lung cancer undergoing radical resection (lobectomy and lymph node dissection, excluding unilateral pneumonectomy) were selected, American Society of Anesthesiologists (ASA) staging I-II. The enrolled patients had normal pulmonary function test, free of obvious cardiovascular and cerebrovascular diseases, central nervous system diseases, serious impairments of hearing, vision or motor, nor history of taking antipsychotic drugs and corticosteroid, chemotherapy and recent infection in the past half year. They were randomly assigned into sevoflurane group (S) and propofol group (P).

All patients received intramuscular injection of 0.5 mg penehyclidine hydrochloride and 0.05–0.08 mg/kg midazolam 30 min before surgery. Patients in the P group were induced by intravenous injection of 1–2 mg/kg propofol, 1–2 μg/kg fentanyl and 0.15 mg/kg cisatracurium, and then maintained by administration of 3–5 mg/kg/h propofol and 0.06–0.12 mg/kg/h cisatracurium via a target-controlled infusion (TCI) system. Patients in the S group were induced by inhalation of 8% sevoflurane and intravenous injection of 1–2 μg/kg fentanyl and 0.15 mg/kg cisatracurium, and then maintained by continuous inhalation of 1.5%-3% sevoflurane and administration of 0.06–0.12 mg/kg/h cisatracurium via TCI system. Patients were injected with 1.0 μg/kg fentanyl intravenously when their blood pressure was higher than the basic blood pressure or the heart rate was higher than 20% during the surgery. The propofol flow rate and sevoflurane concentration were adjusted according to the clinical indicators, hemodynamics and anesthesia depth detector so as to ensure enough anesthesia depth. The use of anesthetic drugs was stopped 10 min before the end of surgery. Anesthesia effectual time, automatic respiratory time, eye opening time, and extubation time were documented.

Subsequently, 5 mL venous blood was obtained before anesthesia induction (T0), 10 min after anesthesia induction (T1), 1 h after the beginning of the operation (T2), 1 h after the end of operation (T3), the 1st day after surgery (T4), the 3rd day after surgery (T5) and the 7th day after surgery (T6) respectively, and anticoagulated with sodium heparin. Meanwhile, mini-mental-state examination (MMSE) [25] was used to evaluate peri- and postoperative cognitive function at different time points from the aspects of orientation, memory, attention, calculation, recall and language. The total scores of 30 meant the best cognitive function, whereas scores < 23 meant cognitive impairment.

### Isolation of peripheral blood mononuclear cells (PBMCs)

The peripheral blood sample was diluted with equal amount of normal saline/ phosphate buffer saline (PBS) and added into lymphocyte separation medium (the blood dilution was slowly added to the surface of lymphocyte separation medium at a uniform speed to ensure clear stratification between blood dilution and lymphocyte separation medium) and centrifuged for 20 min at 500 g at room temperature. The fluid was divided into 4 stratifications from top to bottom: plasma + normal saline/PBS mixture layer, mononuclear cell buffy coat layer, human lymphocyte separation layer and red blood cell layer. The buffy coat layer was carefully absorbed, placed in a new centrifuge tube and mixed with appropriate amount of PBS and centrifuged for 20 min at 500 g at room temperature. The supernatant was removed and precipitation was resuspended in PBS at 4℃ and centrifuged for 5 min at 500 g. The procedure was repeated twice. The supernatant was discarded and precipitation was used to count living cells.

### Flow cytometry

PBMCs used for flow cytometry were pretreated before detection to remove dead cells using fixable viability dye (FVD) that distinguishes dead and living cells.

As for Treg cells, human Treg Detection kit (Miltenyi Biotec, Sunnyvale, CA) was used to identify and quantify CD4^+^CD25^+^Foxp3^+^ Treg cells by FACS analysis. PBMCs were stained with FITC-labeled CD4 antibody and APC-labeled CD25 antibody. After washing, cells were fixed and permeated in fixing/permeating solution, blocked with FcR blocking reagent, stained with PE-labeled Foxp3 antibody with IgG as the isotype control, and placed in the dark at 4℃. FACS data collection and classification was performed using BD FACSVerse flow cytometer (BD Biosciences, San Jose, CA, USA). Raw data were processed using FlowJo software (Treestar, Ashland, OR, USA). The gating of CD4^+^ lymphocytes was detected by forward and side light scatter and CD4 staining. CD4^+^CD25^+^Foxp3^+^Treg subsets in T lymphocyte population were identified and counted. Treg cell number was expressed as the percentage of CD4^+^ cells.

Subsequently, Th17 cells were detected. A previous study has elicited that CD4 molecules in human T cell surface are internalized and reduced surface expression of CD4 molecules is observed after Phorbol 12-myristate 13-acetate (PMA)/Ionomycin stimulation [26], making it difficult to gate CD4^+^ T cells in flow cytometry. Therefore, surrogate markers such as CD3^+^ [27] or CD3^+^CD8^−^ [28] are often used in detection of Th in human whole blood. In this study, we defined CD4^+^ T cells by staining CD3 and CD8 to circle CD3^+^CD8^−^ cells in detection of Th in human peripheral blood. Specifically, the PBMCs were isolated. The 6-well plate was added with 1 mL cell suspension, 50 ng PMA, 1 μg ionomycin and 0.7 μL monensin per well, and then cultured at 37℃ for 6 h. After centrifugation, the cells were transferred to the flow detection tube, suspended by PBS, incubated with antibodies CD3 and CD8, and supplemented with fixed membrane penetrating agent. After washing and resuspending, the cells were added with antibody interleukin (IL)-17A with IgG acting as the isotype control, and then cells were placed in the dark at 4℃, followed by the detection on the flow cytometry.

The antibodies used were following: FITC-labeled mouse monoclonal antibody CD4 (ab18281, Clone MEM-241, 0.5 µg/10^6^ cells, Abcam, Cambridge, MA, USA), APC-labeled mouse monoclonal antibody CD25 (0.125 µg, ab267381, Clone BC96, Abcam), PE-labeled mouse monoclonal antibody Foxp3 (0.25 µg, ab218773, Clone MF23 Abcam), PE-labeled mouse monoclonal antibody IL-17A (eBIO64DEC17, 0.25 µg, Clone eBio17B7, Thermo Fisher Scientific, Shanghai, China), FITC-labeled mouse monoclonal antibody CD3 (1 µg, ab34275, Clone UCHT1, Abcam), APC-labeled mouse monoclonal antibody CD8 (ab26004, Clone MEM-31, 0.5 µg/10^6^ cells, Abcam), FITC-labeled mouse IgG1 isotype (ab91356, Clone B11/6, 20 µL/10^6^ cells, Abcam), PE-labeled mouse IgG2b isotype control (0104–09, Clone A-1, 10 µL/10^6^ cells, AmyJet Scientific, Wuhan, China), PE-labeled rat IgG2a isotype (48–4321-82, Clone eBR2a, 0.25 µg, Thermo Fisher Scientific), APC-labeled mouse IgG1 isotype control (0102–11, Clone 15H6, 10 µL/10^6^ cells, AmyJet Scientific), APC-labeled mouse IgG2a isotype (ab91364, Clone B12/8, 20 µL/10^6^ cells, Abcam). Th17/Treg ratio in peripheral blood = CD3^+^CD8^−^IL-17^+^ cells/CD4^+^CD25^+^Foxp3^+^ cells.

### Enzyme-linked immunosorbent assay (ELISA)

The levels of IL-17 (SM1700; Sensitivity: 5 pg/mL; Assay Range: 10.9—700 pg/mL), IL-10 (S1000B; Sensitivity: 3.9 pg/mL; Assay Range: 7.8–500 pg/mL), and transforming growth factor (TGF)-β (SB100B; Sensitivity: 15.4 pg/mL; Assay Range: 31.2–2000 pg/mL) in peripheral blood were measured based on the instructions of ELISA kits (R&D Systems, Minneapolis, MN, USA).

### Quantitative real-time polymerase chain reaction (qRT-PCR)

Total RNA in peripheral blood was extracted using a TRIzol reagent (Invitrogen, Carlsbad, CA, USA). The purity of extracted RNA was measured using ultraviolet analysis and formaldehyde deformation electrophoresis. Fluorescent quantitative PCR was performed on the instructions of qRT-PCR kit (Thermofisher scientific, Shanghai, China). Primers (Table 1) were designed and synthesized by Sangon Biotech Co., Ltd, (Shanghai, China). Glyceraldehyde-3-phosphate dehydrogenase (GAPDH) acted as the internal reference. The amplification curve and dissolution curve were confirmed after reaction. The relative expression of genes was calculated by 2^−ΔΔCt^ method.

**Table 1:** Primer sequences for qRT-PCR

Gene	Sequence (5′-3′)
GAPDH	F: 5′-GGGAGCCAAAAGGGTCAT-3′
	R: 5′-GAGTCCTTCCACGATACCAA-3′
RORγt	F: 5ʹ-GAAGTGATCCCTTGCAAGAT-3ʹ
	R: 5ʹ-CTTTGACAGCCCCTCAGGGG-3ʹ
Foxp3	F: 5′-CAACCCAAGGCCAGCTAAGC-3′
	R: 5′-GGCAGGGGTTGGAGCACTTG-3′

### ***CD4***^+^***T cell culture and treatment***

The peripheral blood was collected before surgery. PBMCs from peripheral blood were collected as aforementioned and filtered using a 70-μm filter, added with 10 mL Ficoll-Paque and centrifuged for 30 min at 400 g at 20℃. The cloud-like lymphocyte layer was sorted using CD4+ magnetic beads (130–045-101, Miltenyi Biotec, Bergisch Gladsbach, Germany). Lymphocytes were isolated from peripheral blood. CD4^+^ T cells were obtained using immunomagnetic beads and then resuspended in RPMI-1640 medium containing 10% fetal bovine serum (FBS). After 24-h culture, the cell viability was detected using Trypan blue staining.

The cells were assigned into: blank group (CD4^+^ T cells), P1 group (CD4^+^ T cells + 0.3 μg/mL propofol), P2 group (CD4^+^ T cells + 3 μg/mL propofol), P3 group (CD4^+^ T cells + 30 μg/mL propofol), B1 group [CD4^+^ T cells + 0.3 μg/mL propofol + 100 μM bicuculline (GABAA receptor antagonists) (MedChemExpress, LLC, NJ, USA)], B2 group (CD4^+^ T cells + 3 μg/mL propofol + 100 μM bicuculline), and B3 group (CD4^+^ T cells + 30 μg/mL propofol + 100 μM bicuculline). Two tubes were utilized in each group, of which one tube used to detect Treg cells and the other tube added with 25 ng/mL PMA and 1 μg/mL ionomycin to detect Th17 cells. Tubes in each group were incubated at 37℃ with 5% CO_2_ for 4 h.

### Lung cancer cell culture and treatment

Human lung cancer cell line NCI-H1975, Lewis lung carcinoma cell line 3LL and normal lung epithelial cell line Beas-2B purchased from American Type Culture Collection were cultured in Dulbecco’s modified Eagle’s medium (DMEM) containing 10% FBS, 100 Ku/L penicillin and 100 Ku/L streptomycin at 37℃ with 5% CO_2_. NCI-H1975 and Beas-2B cells at logarithmic growth phase were washed with PBS twice to remove serum, detached with 0.25% trypsin for 3–5 min, and added with normal culture medium to terminate detachment. Cells on the bottle wall were gently blown down, centrifuged at 100 g for 5 min before collection, and added with normal culture medium to prepare single cell suspension. Until 70–80% confluence under normal culture conditions, NCI-H1975 and Beas-2B cells were treated with CD4^+^ T cell conditioned culture medium (blank group), CD4^+^ T cell conditioned culture medium pretreated with 30 μg/mL propofol (P group), or CD4^+^ T cell conditioned culture medium pretreated with 30 μg/mL propofol + 100 μM bicuculline (B group), and cultured for another 24 h for subsequent experimentation.

### Cell counting kit-8 (CCK-8) assay

Aforementioned 6-well plate was incubated for 96 h at 37℃ with 5% CO_2_. The cell viability was detected based on the instructions of CCK-8 kit (C0038, Beyotime Biotechnology Co., Ltd, Shanghai, China). NCI-H1975 cells or 3LL cells were seeded into the 96-well plate at 5000 cells/well. Then the culture medium was replaced with 100 μL CCK-8 solution (serum-free DMEM containing 90 μL and 10 μL CCK-8 solution). The optical density at a wavelength of 450 nm of each well was evaluated using a microplate reader (Thermo Fisher Scientific).

### Transwell assays

For cell migration assay, cells were collected, added with serum-free culture medium (0.1% bovine serum albumin), resuspended to 1 × 10^5^ cells/mL and added to the apical chamber. The basolateral chamber was supplemented with DMEM containing 10% FBS. Then the Transwell chamber was incubated at 37℃ with 5% CO_2_ for 24 h. After being washed by PBS, the filter membrane was fixed with 0.5% glutaraldehyde, stained with crystal violet and incubated at 37℃ with 5% CO_2_ for 24 h. The experimental steps of cell invasion assay were mostly the same as those above, but it should be noted that Transwell chamber was pre-coated with matrix. Following 24-h incubation, cells in apical chamber were removed with cotton swabs and cells in the basolateral chamber were fixed with 4% paraformaldehyde, stained with crystal violet (Sigma-Aldrich, St. Louis, MO, USA) and observed and photographed under a microscope (Olympus CKX41, Tokyo, Japan). Cells in randomly selected 5 fields of vision were observed, photographed and counted.

### Immunocytochemistry

Cells were incubated in a culture dish with a cover glass, and the culture medium was removed when the cover glass was full of cells. Then, cells were washed with PBS, fixed with 4% formaldehyde for 15 min, permeabilized with 0.1% Triton X-100 for 5 min and then blocked with 1% BSA/10% normal goat serum/0.3 M glycine in 0.1% PBS-Tween for 1 h. The cells were then incubated overnight at 4℃ with E-cadherin antibody (1:100, ab194982, Abcam). Subsequently, cells were observed under a fluorescence microscope (Olympus CX23, Tokyo, Japan). Image was taken with an Operetta High Content Analysis System (Perkin Elmer Foster City, CA, USA).

### Xenograft tumors in nude mice

Totally 60 healthy aged C57BL/6 mice (aged 18–24 months) were obtained from Medicilon Pharmaceutical Technology (Shanghai) Co., Ltd [SYXK (Shanghai) 2018-0025, Shanghai, China]. A total of 1 × 10^6^ lung cancer cells (NCI-H1975 or 3LL) were mixed with matrigel at the ratio of 1:1, and then subcutaneously injected into each mouse via the caudal vein. The general condition of mice was observed after surgery. The CT images of mice's chest were scanned with a small animal irradiator to observe whether there were pulmonary space-occupying lesions. On the 12th day after the tumor transplantation, lung tumors in mice were removed. Before the surgery, the blood was collected from the orbit. After the surgery, the mouse survival in each group was observed. All mice were euthanized with intraperitoneal injection of pentobarbital (800 mg/mL) on the 45th day of tumor transplantation [29, 30]. The blood was collected from orbit again before euthanasia. The lung and liver of mice in each group were removed to count the obvious tumor nodules for evaluation of metastasis to the lung and liver, and then immediately fixed with paraformaldehyde and embedded in paraffin.

The mice were assigned into: sevoflurane (S) group [10 mice injected with NCI-H1975 cells and 10 mice injected with 3LL cells, tumors were resected under sevoflurane anesthesia (Sevoflurane 3% in 50% oxygen-air mixture [31] and mice were injected with PBS via the tail vein 24 h before surgery], propofol (P) group [10 mice injected with NCI-H1975 cells and 10 mice injected with 3LL cells, tumors were resected under propofol anesthesia (Sevoflurane 3% in 50% oxygen-air and intravenous propofol; bolus of 5 mg/kg over 30–60 s [31] and mice were injected with PBS via the tail vein 24 h before surgery], propofol + biculline (P + B) group (10 mice injected with NCI-H1975 cells and 10 mice injected with 3LL cells, tumors were resected under propofol anesthesia and mice were injected with 5 mg/kg bicuculline via the tail vein 24 h before surgery) [32]. The period from induction to cessation of anesthesia was standardized at 30 min for all animals.

### Histological staining

Hematoxylin & eosin (HE) staining and immunohistochemical staining were adopted to observe the metastasis of lung cancer and protein expression. The deparaffinized sections were stained with hematoxylin and eosin (Solarbio, Beijing, China), and then observed under the optical microscope (Leica, Solms, Germany).

### Statistical analysis

SPSS 21.0 software (SPSS Inc., Chicago, IL, USA) was applied for data analysis. Shapiro–Wilk test (W test) showed that the data were in normal distribution. Data are expressed as mean ± standard deviation. The *t* test was adopted for analysis of comparisons between two groups. One-way or two-way analysis of variance (ANOVA) was employed for the comparisons of multiple groups and Tukey’s multiple comparisons test was applied for post hoc test. The *p* value was obtained by a two-tailed test and *p* < 0.05 indicated a significant difference.

## Results

### Propofol anesthesia maintained the balance of Th17/Treg cell in the elderly

There is evidence that accumulation of immunosuppressive Treg lymphocytes participates in sustaining immune self-tolerance and homeostasis. Compared with healthy people, Th17/Treg ratio is decreased in elderly lung cancer patients, and Th17/Treg cell balance is compromised, leading to reduction of anti-tumor immune response [13]. Surgical trauma interferes immune function and inhibits postoperative adaptive immune response and anti-tumor immunity, whereas increased Treg cell expression produces significant effect on mediating this immunosuppression [12]. In order to explore the action of different anesthetic drugs on Th17/Treg cell balance in patients undergoing radical resection of lung cancer, altogether 36 elderly patients with lung cancer were collected. Among them, 18 patients were anesthetized with sevoflurane (S) and 18 patients were anesthetized with propofol (P). There was no statistical difference between the two groups in age and gender. We firstly compared the conditions of anesthesia and analepsia, and found no significant difference in anesthesia time, automatic respiratory time, eye opening time, and extubation time between these two groups (all *p* > 0.05) (Table 2). Moreover, there was no noticeable difference in MMSE scores a day prior to surgery based on detection before anesthesia induction (T0), 10 min after anesthesia induction (T1), 1 h after the beginning of the operation (T2), 1 h after the end of operation (T3), the 1st day after surgery (T4), the 3rd day after surgery (T5) and the 7th day after surgery (T6), and MMSE scores of both groups were decreased 6–7 h after surgery and showed an upward trend compared with perioperative ones, whereas MMSE scores showed no remarkably difference between these two groups, and all cognitive dysfunctions of all patients disappeared 7 days after operation (all *p* > 0.05, Table 3).

**Table 2:** Anesthesia and aweakening condition of lung cancer patients with different anesthesia methods

	Sevoflurane (S) N = 18	Propofol (P) N = 18	*p*
Age	72.5 ± 5.6	73.1 ± 6.2	0.76
Gender (male/female)	12/6	13/5	0.131
Anesthesia time (min)	210.05 ± 35.06	203.11 ± 36.21	0.563
Automatic respiratory time (min)	10.64 ± 3.17	11.52 ± 3.22	0.414
Eye opening time (min)	15.70 ± 3.55	16.35 ± 3.72	0.115
Extubation time (min)	19.99 ± 3.45	21.03 ± 3.69	0.389

**Table 3:** MMSE of lung cancer patients with different anesthesia methods

	Sevoflurane (S) N = 18	Propofol (P) N = 18	*p*
T0	28.98 ± 2.35	28.59 ± 2.08	0.602
T1	28.03 ± 2.57	28.51 ± 2.33	0.561
T2	20.12 ± 2.31	20.43 ± 2.26	0.687
T3	21.64 ± 2.17	21.52 ± 2.22	0.871
T4	23.08 ± 2.37	23.42 ± 2.44	0.674
T5	26.49 ± 2.50	26.03 ± 2.98	0.619
T6	28.64 ± 2.33	28.70 ± 2.41	0.940

**Fig. 1:**
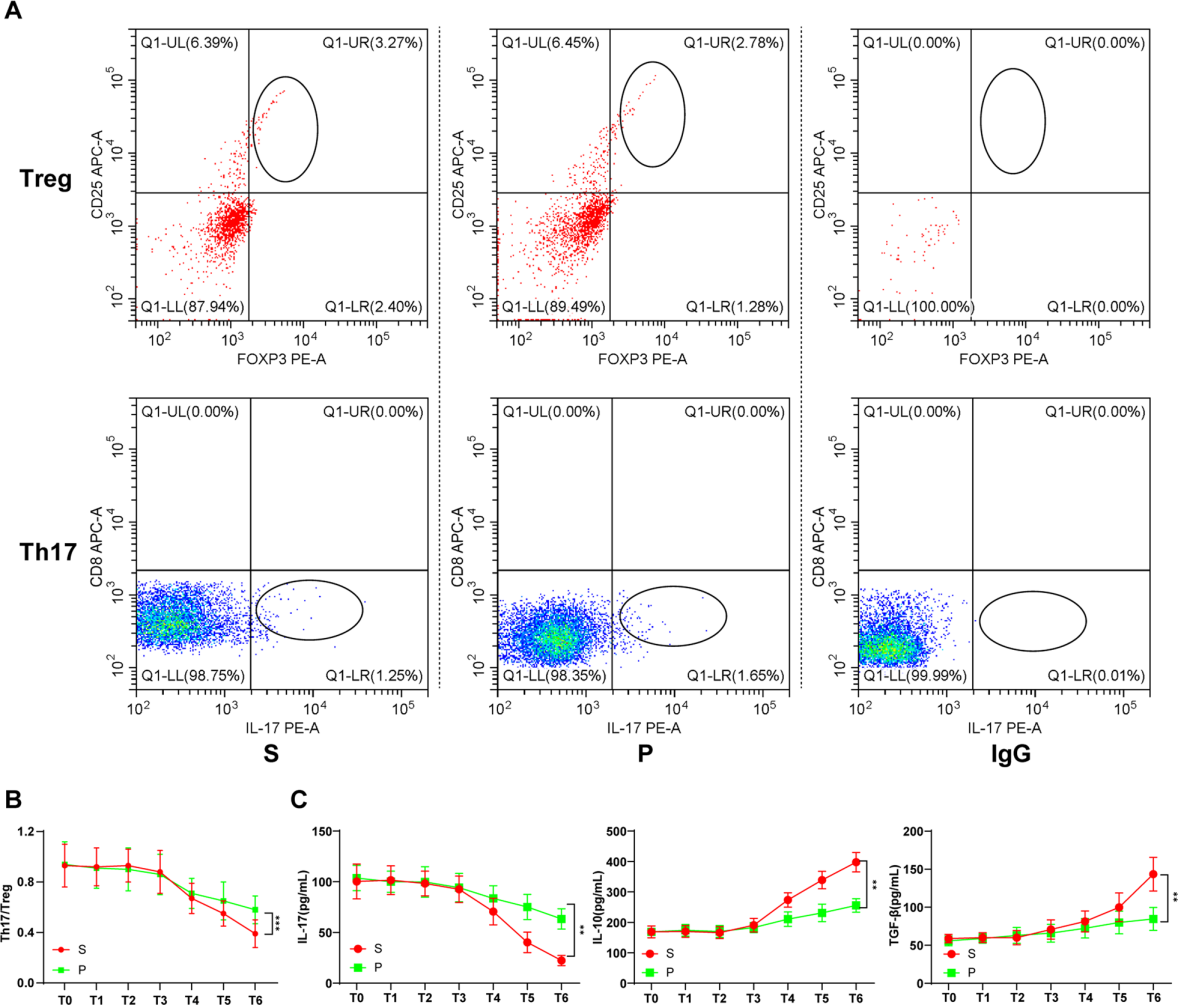
Effects of different anesthesia on the ratio of Th17/Treg cell in peripheral blood of lung cancer patients at different ages. **A** The flow scatter diagram of T6; **B** Th17/Treg ratio during perioperative period; **C** Levels of Th17-related cytokine (IL-17) and Treg-related cytokines (IL-10, TGF-β) during perioperative period were detected by ELISA. In brief, 5 mL venous blood was taken before anesthesia induction (T0), 10 min after anesthesia induction (T1), 1 h after the beginning of the operation (T2), 1 h after the end of operation (T3), the 1st day after operation (T4), the 3rd day after operation (T5) and the 7th day after operation (T6) respectively. Treg cells were screened from CD4^+^ T cells by CD25/Foxp3, with IgG acting as the isotype control. PBMCs cells were incubated with antibody CD3/CD8 and then added with fixed membrane penetrating agent. After washing and resuspending, PBMCs cells were added with antibody IL-17A to screen Th17 cells, with IgG acting as the isotype control. All experiments were repeated three times. Data in panels B/C were analyzed using one-way ANOVA, followed by Tukey's multiple comparisons test, ***p < 0.001

Subsequently, venous blood was collected from patients before anesthesia induction (T0), 10 min after anesthesia induction (T1), 1 h after the beginning of the operation (T2), 1 h after the end of operation (T3), the 1st day after surgery (T4), the 3rd day after surgery (T5) and the 7th day after surgery (T6) respectively. PBMCs were subsequently isolated, followed by flow cytometry to detect CD4^+^CD25^+^Foxp3^+^Treg cells (CD4^+^ cells were used for sorting) and CD3^+^CD8^−^IL-17^+^Th17 cells (CD3^+^CD8^−^ cells were used for sorting) (as shown in Additional file 1: Figs. S1, S2), and the Th17/Treg ratio in peripheral blood of elderly patients undergoing lung cancer surgery after different anesthesia was calculated. As shown in Table 4, Th17/Treg ratio in the peripheral blood of elderly patients undergoing lung cancer surgery after different anesthesia (T3–T6) was decreased, suggesting anti-tumor immune effect on elderly lung cancer patients was further decreased by surgical trauma. Compared with sevoflurane, propofol-treated elderly lung cancer patients had increased Th17 accumulation (1.65% vs. 1.25%), and decreased Treg cells (2.78% vs. 3.27%) after operation (Fig. 1A), thus relieving the decrease of postoperative Th17/Treg ratio, and better maintaining the balance of Th17/Treg cell (flatter curve) (Fig. 1B, *p* < 0.001). In addition, we detected the levels of Th17-related cytokine (IL-17) and Treg-related cytokines (IL-10, TGF-β) in the peripheral blood of elderly lung cancer patients after operation, and found consistent tendency with Th17 and Treg cells. The level of Th17-related cytokine (IL-17) was augmented and the levels of Treg-related cytokines (IL-10, TGF-β) were diminished in the peripheral blood of propofol-anesthetized elderly lung cancer patients after operation (Fig. 1C, all *p* < 0.01). These results elicited that propofol treatment sustained Th17/Treg cell balance in elderly patients undergoing radical resection of lung cancer by increasing Th17 cells and decreasing Treg cells to elevate Th17/Treg ratio.

**Table 4:** Th17/Treg ratio in the peripheral blood of lung cancer patients with different anesthesia methods

	Sevoflurane (S) N = 18	Propofol (P) N = 18	*p*
T0	0.93 ± 0.17	0.94 ± 0.18	> 0.99
T1	0.92 ± 0.15	0.91 ± 0.16	> 0.99
T2	0.93 ± 0.13	0.90 ± 0.17	> 0.99
T3	0.88 ± 0.17	0.86 ± 0.16	> 0.99
T4	0.67 ± 0.12	0.71 ± 0.12	0.97
T5	0.55 ± 0.10	0.65 ± 0.15	0.25
T6	0.39 ± 0.11	0.58 ± 0.11	0.00*

*p < 0.05

### Propofol affected the balance between Th17/Treg cells through GABAA receptor

**Fig. 2:**
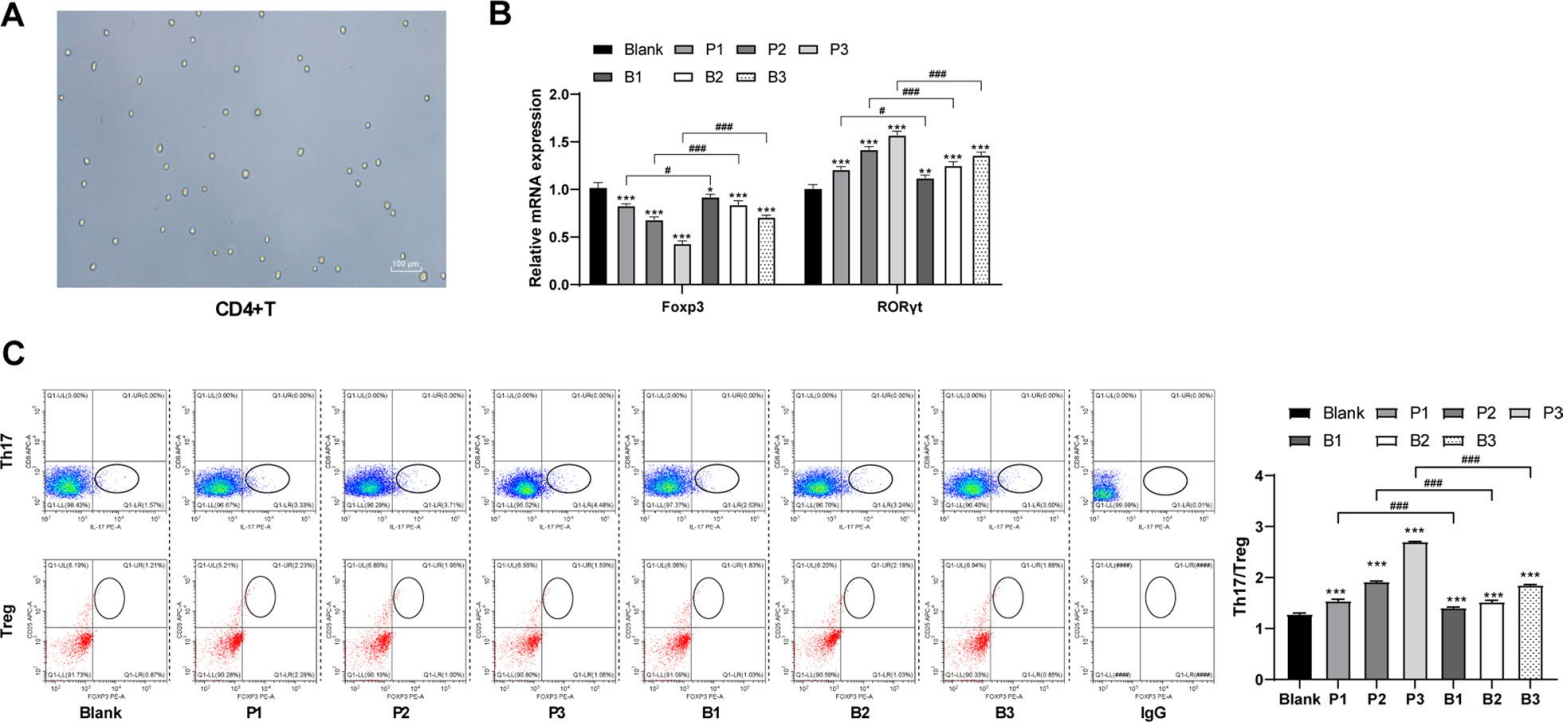
Propofol affected the balance between Th17/Treg cells through GABAA receptor. **A** Results of trypan blue staining on CD4^+^ T cells showed that the dead cells was stained blue, while the living cells was not stained; **B** Expressions of RORγt and Foxp3 in CD4^+^ T cells were detected by qRT-PCR; **C** Ratio of Th17/Treg cell treated with propofol at different concentrations was detected using flow cytometry; the concentrations of P1, P2 and P3 were 0.3 μg/mL, 3 μg/mL and 30 μg/mL respectively; B1, B2 and B3 represented that 100 μM of bicuculline (GABAA receptor antagonist) was added on the basis of P1-P3. All experiments were repeated three times. Data in panel B/C were analyzed using one-way ANOVA, followed by Tukey's multiple comparisons test. Compared with the blank group, **p < 0.01, ***p < 0.001; compared with the inter groups, ^#^*p* < 0.05, ^##^*p* < 0.01, ^###^*p* < 0.001

The peripheral blood of patients with lung cancer was collected and CD4^+^ T cells were obtained using immunomagnetic beads. The cell activity was detected using Trypan blue staining (Fig. 2A). The cells were treated with different concentrations of propofol and then it was found that the level of Th17 cell-related transcription factor retinoid-related orphan receptor γt (RORγt) increased significantly while the level of Treg cell-related transcription factor Foxp3 decreased, and the ratio of Th17/Treg cell increased with the increase of propofol concentrations (all *p* < 0.05). It has been reported that the mechanism of propofol anesthesia is mainly related to GABAA receptor [33], so we speculated that the effect of propofol on the ratio of Th17/Treg cell may also be concerned with GABAA receptor. Therefore, we treated CD4^+^ T cells with bicuculline, a GABAA receptor antagonist, and then found that the abilities of propofol to increase the Th17/Treg cell ratio were impaired (all *p* < 0.05) (Fig. 2B, C). These findings indicated that propofol affected the balance between Th17/Treg cells through the GABAA receptor.

### Propofol inhibited the invasion of lung cancer cells through GABAA receptor

**Fig. 3:**
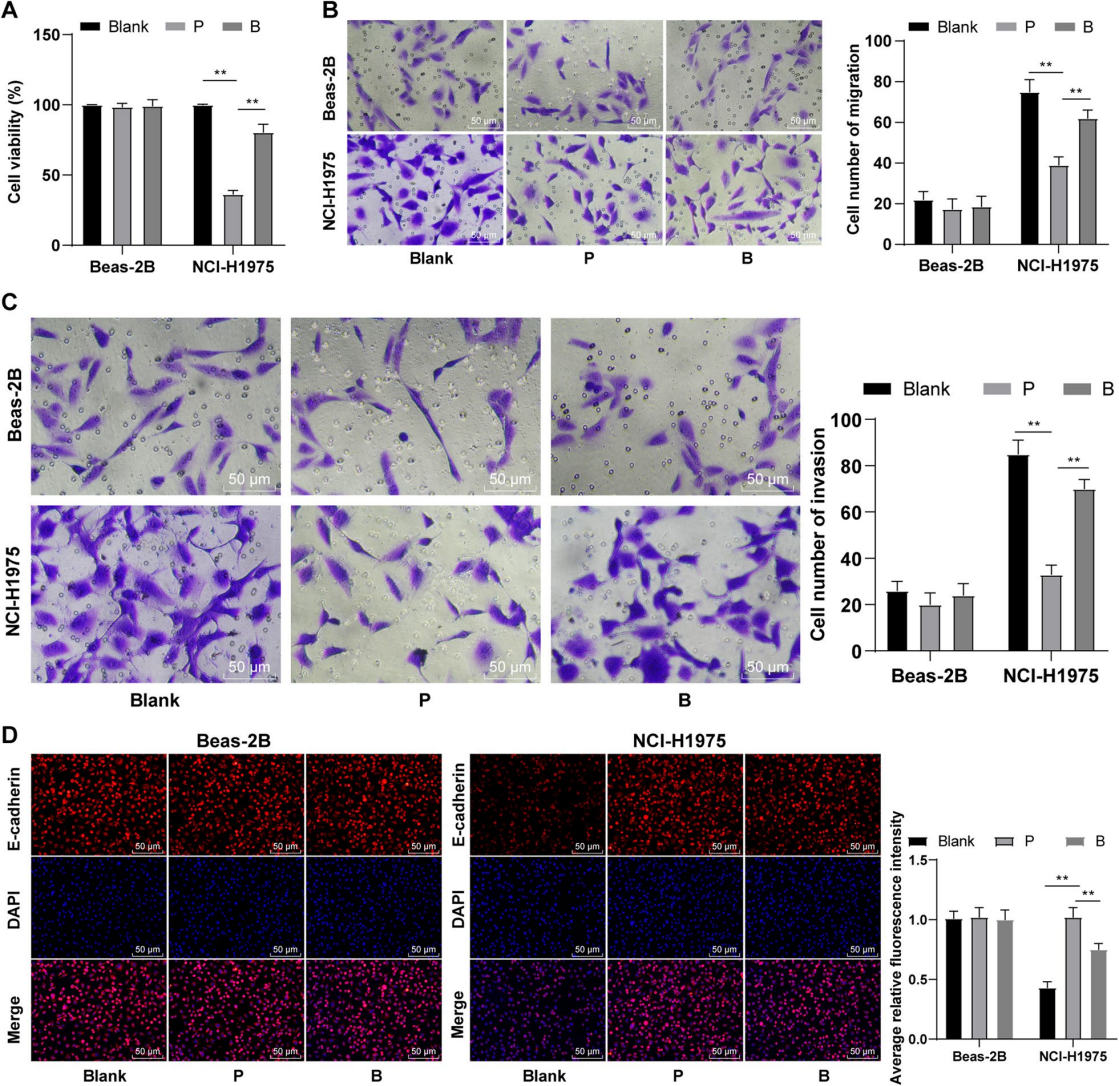
Propofol inhibited the migration and invasion of lung cancer cells through GABAA receptor. **A** Effect of 30 μg/mL propofol on lung cancer cell viability was measured by CCK-8 assay; **B** Cell migration were detected using Transwell assay; **C** Cell invasion was detected using Transwell assay; **D** Positive rate of E-cadherin protein was detected by immunocytochemistry and the stronger red fluorescence meant the higher positive rate. All experiments were repeated three times. Data were analyzed using independent sample *t* test, **p < 0.01, ***p < 0.001

Propofol has been shown to reduce lung metastasis in the mouse model of breast cancer surgery [31], and the recurrence and metastasis of cancer after surgery are related to the immune ability of patients. So we cultured human lung cancer cell line NCI-H1975 or normal lung epithelial cell line Beas-2B with CD4^+^ T cells (blank group) or CD4^+^ T cells pretreated with 30 μg/mL propofol (P group), respectively. CCK-8 assay showed that the viability of lung cancer cells cultured with CD4^+^ T cell conditioned medium pretreated with propofol decreased significantly (*p* < 0.05, Fig. 3A). Transwell assays showed that the invasion and migration of lung cancer cells cultured with CD4^+^ T cell conditioned medium pretreated with propofol decreased notably. The E-cadherin protein expression in cells increased significantly. However, propofol had no effect on the invasion and migration of normal pulmonary epithelial cell line Beas-2B (all *p* < 0.001) (Fig. 3B–D). Briefly, propofol inhibited the invasion of lung cancer cells. Moreover, we subsequently detected the function of different doses of propofol on viability, invasion and migration of lung cancer cells. The result showed that propofol affected lung cancer cell invasion in a dose-dependent manner, which was also verified in human lung cancer cell line 3LL (Additional file 1: Fig. S3). Furthermore, we set up an additional group using NCI-H1975/Beas-2B cells treated with CD4^+^ T cell conditioned medium pretreated with 30 μg/mL propofol + 100 μM bicuculline (B group), and observed a reversal effect of propofol on inhibiting lung cancer viability, invasion, and migration by addition of bicuculline (all *p* < 0.001, Fig. 3A–D), which suggested that propofol affected lung cancer cell migration and invarion by affecting Th17/Treg cell balance through GABAA receptor.

### Propofol maintained balance of Th17/Treg cells in aged mice with lung cancer after surgery

**Fig. 4:**
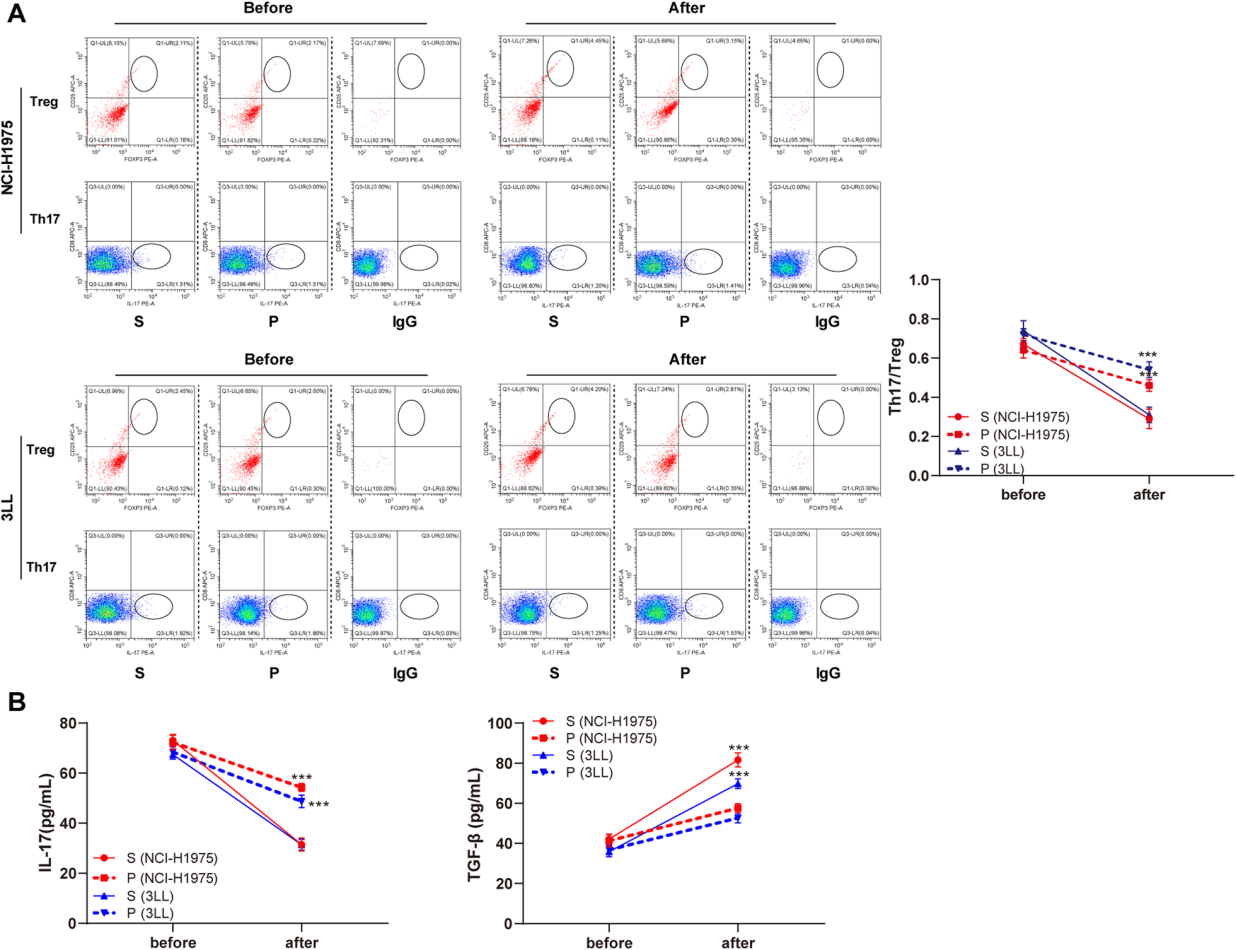
Propofol maintained balance of Th17/Treg cells in aged mice. **A** Ratio of Th17/Treg cell in peripheral blood of mice before and after surgery was measured by flow cytometry; **B** Expressions of IL-17 and TGF-β in peripheral blood of mice before and after surgery were measured by ELISA. All experiments were repeated three times. N = 10. Data were analyzed using two-way ANOVA, followed by Tukey's multiple comparisons test, ***p < 0.001

The mice aged 18–24 months were selected to establish the model of lung cancer in situ, and the tumors were removed on the 12th day after injection of tumor cells. The change of Th17/Treg cell ratio in propofol anesthetized mice was much less prominent than that in sevoflurane anesthetized mice. In addition, the sevoflurane anesthetized mice showed significantly decreased IL-17 level and increased TGF-β level, but there were no such obvious changes in propofol anesthetized mice (all *p* < 0.001) (Fig. 4A, B).

### Inhibition of GABAA receptor offset propofol’s effect on Th17/Treg cell balance

**Fig. 5:**
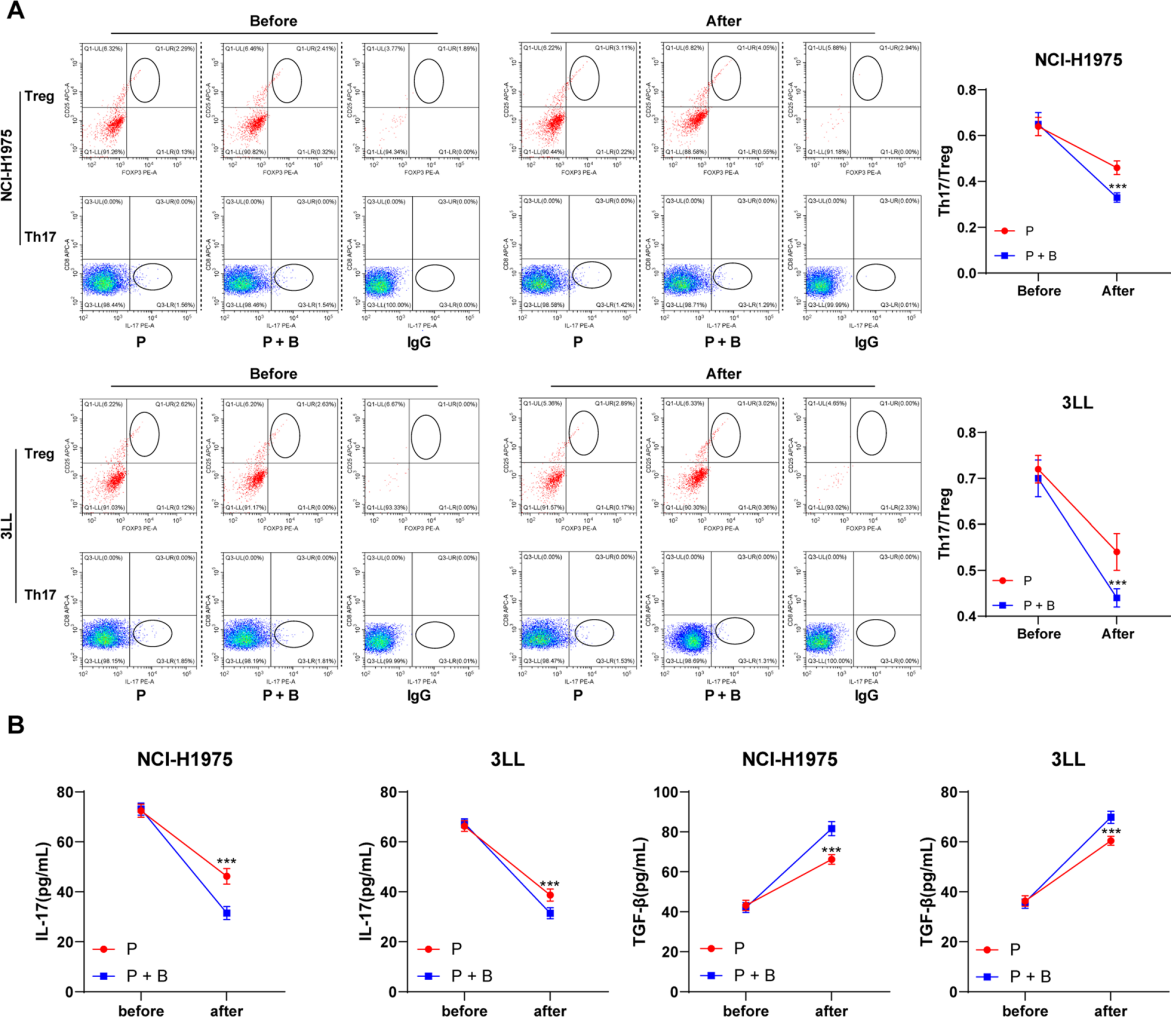
Propofol regulated the balance of Th17/Treg cell in mice with lung cancer before and after surgery through GABAA receptor. **A** Ratio of Th17/Treg cell in peripheral blood of mice before and after surgery was measured by flow cytometry; **B** Expressions of IL-17 and TGF-β in peripheral blood of mice before and after surgery were measured by ELISA. All experiments were repeated three times. N = 10. Data were analyzed using two-way ANOVA, followed by Tukey's multiple comparisons test, ***p < 0.001

Subsequently, we treated mice with GABAA receptor antagonist, and found that propofol’s effect on the balance of Th17/Treg cell in aged mice disappeared (Fig. 5A, B), indicating that propofol regulated the balance of Th17/Treg cell in lung cancer mice before and after surgery through GABAA receptor.

### Propofol reduced the metastasis of lung cancer in mice after surgery

**Fig. 6:**
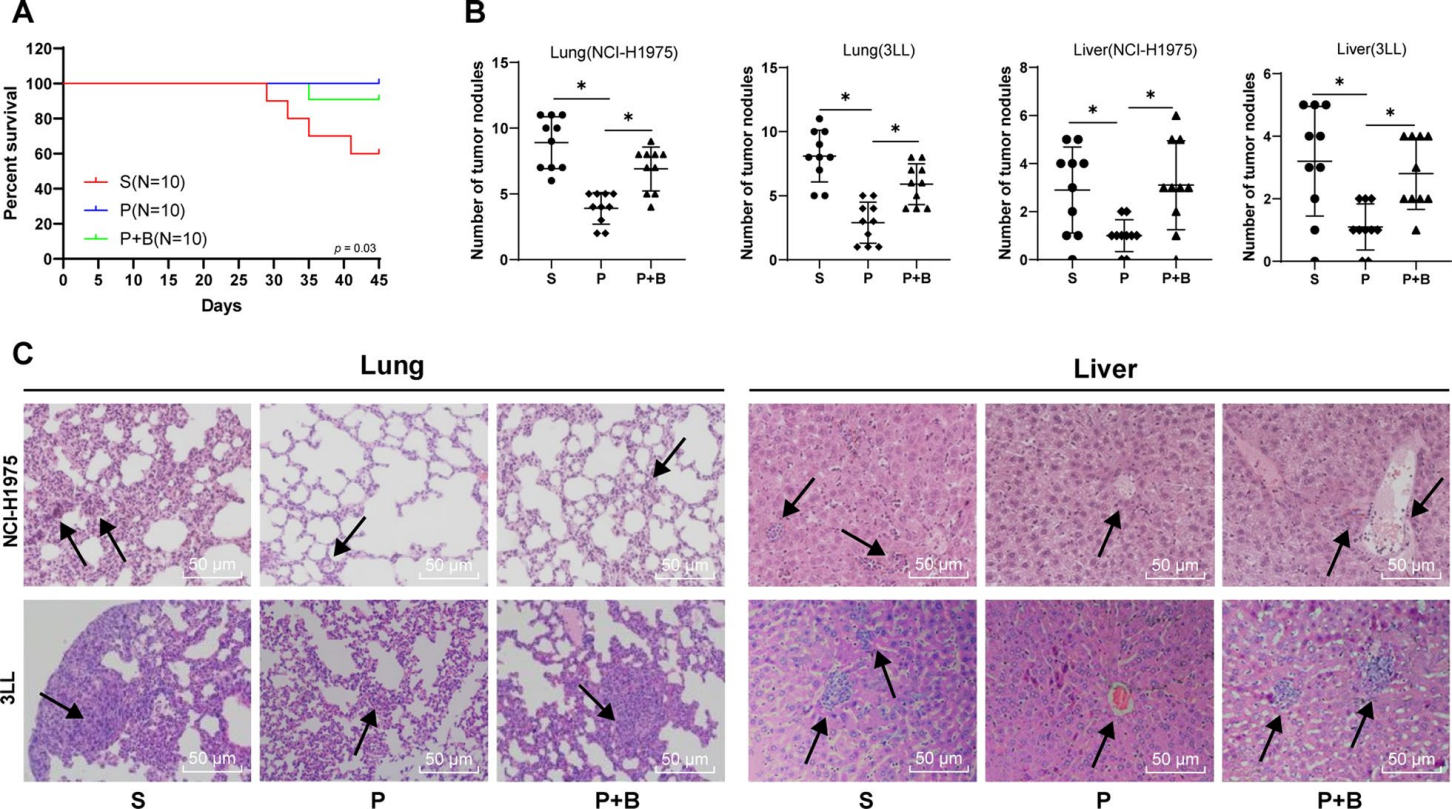
Propofol reduced the metastasis of lung cancer in mice after operation. **A** Survival curve of mice after lung cancer resection on the 12th day; **B** Quantitative analysis of tumor nodules in lung and liver; **C** The histopathology of the lung and liver of mice was observed using H&E staining. All experiments were repeated three times. N = 10

We observed the survival rate of mice after the surgery (Fig. 6A). On the 45th day after injection of tumor cells (on the 33rd day after tumor resection), all surviving mice were euthanized and their lung and liver were taken. Tumor nodules in the lung and liver were counted (Fig. 6B) to evaluate metastasis of breast cancer to lung and liver. Meanwhile, pathological changes were observed by HE staining (Fig. 6C).The mice in the S group showed lower survival rate compared with the mice in the P group. Although cancer metastasis was observed in liver and other organs (0.2–3 cm nodule size, 9 nodules), the metastasis rate of mice in the P group was significantly lower than that in the S group. The results of the bicuculline and propofol combined treatment group (P + B group) were similar to those of the S group. The P + B group exhibited a decreased survival rate and an increased metastatic rate compared with the P group. It was suggested that propofol anesthesia had a good effect on reducing the metastasis of lung cancer and increasing the survival rates of mice with lung cancer after surgery.

## Discussion

Perioperative anesthesia may aggravate immunosuppression in cancer patients with immune deficiency, especially for the elderly [34]. It is reported that inhalation anesthesia such as sevoflurane may induce immunosuppression and activate inflammation [35], while propofol can reduce immunosuppression and recurrence of some cancers via decreasing the neuroendocrine response induced by surgery [36]. Imbalance of Th17/Treg cell can contribute to the initiation and progression of some diseases related to autoimmune, infection and tumors [37, 38]. However, the relationship between propofol and Th17/Treg cell balance in lung cancer after surgery remains unclear till now. In view of this, this study investigated the effect of propofol on the balance of Th17/Treg cell in elderly patients with lung cancer after radical resection.

The significant roles of Th17 cells and Treg cells in human immune and cancer processes have been unveiled [39]. In this study, 36 elderly patients with lung cancer were randomly anesthetized with sevoflurane or propofol, and then the ratio of Th17 cells and Treg cells in peripheral blood of each patient was detected using flow cytometry. Our study found that propofol anesthesia better maintained the balance of Th17/Treg cell during perioperative period of elderly patients with lung cancer. A former study has noticed increased Treg cells and Foxp3 expression, decreased Th17 cells and RORγt expression and Th17/Treg ratio, and negative correlation of Th17/Treg to cancer stages in the peripheral blood of elderly lung cancer patients, compared with healthy people [13]. All these findings suggest that Th17/Treg ratio is decreased and Th17/Treg balance is compromised in elderly lung cancer patients, contributing to a decrease in anti-tumor immune effects. Th2 lymphocyte expression increases in trauma and surgery-associated physical injury, leading to impairment of CMI, and increase in Treg cell expression plays a fundamental role in mediating the immunosuppression [12]. Our results demonstrated a downward trend of Th17/Treg ratio in the peripheral blood of elderly lung cancer patients/mice after surgery, indicating that surgical trauma further reduced the anti-tumor immune effect on elderly lung cancer patients, whereas propofol anesthesia slowed down the declining of Th17/Treg ratio and sustained Th17/Treg cell balance. To our knowledge, our study was the first to demonstrate that propofol participated in the balance of Th17/Treg in lung cancer cells.

CD4^+^ T cells are members of effector T cells and have influence on infection-related diseases and tumor immunity [40], which can differentiate into Th17 cells and Treg cells [41]. RORγt and Foxp3 are the transcription factors of Th17 and Treg, respectively, whose expressions directly affect the balance of Th17/Treg cell [42]. Then, we collected the peripheral blood of patients with lung cancer in vitro and obtained CD4^+^ T cells by immunomagnetic beads method. The CD4^+^ T cells treated with propofol showed an increased RORγt expression and a decreased Foxp3 expression, implying that the ratio of Th17/Treg cell elevated with the increase of propofol concentrations. Current studies regarding propofol mediating Th17/Treg cell balance are rare. A recent study reported that propofol maintained Th17/Treg cell balance and reduced inflammation in rats with traumatic brain injury via the miR-145-3p/NFATc2/NF-κB axis [43], proposing a possible regulatory mechanism of propofol in Th17/Treg cell balance. Our study initiated that propofol affected postoperative Th17/Treg cell balance in elderly lung cancer patients, yet the mechanism behind remains unknown. According to previous studies, human PBMC population, including lymphocytes, can express functional GABAA receptors that are able to regulate immune responses [44]. GABAA receptor agonist, a new treatment method, enhances CD8 and CD4 Treg response and limits inflammation mediated by Th17 and Th1 [45]. Moreover, it has been reported that the analgesic mechanism of propofol is associated with GABAA receptor [33]. These results elucidated that propofol might affect Th17/Treg cell balance by increasing GABAA receptor activity on CD4^+^ T cell membrane. After GABAA antagonists were added to CD4^+^ T cells, propofol’s ability to increase Th17/Treg cell ratio was inhibited. These results indicated that propofol affected the balance between Th17/Treg cells through GABAA receptor. Moreover, propofol is proved to exert tumor suppressive effect in vitro and in vivo [46]. Propofol is reported to suppress the invasion and migration of endometrial cancer cells [47]. Accordingly, this study found that the invasion and migration of lung cancer cells treated with propofol decreased notably with the increase of propofol concentrations in a dose-dependent manner.

Thereafter, we performed animal experiments and established the model of lung cancer in situ in aged mice. Th17 cells secrete IL-17, which further enhances inflammation and tumorigenesis [48, 49]. Treg cells secrete TGF-β, which inhibits anti-tumor immune responses [37]. The propofol anesthetized mice had an increased IL-17 expression and a decreased TGF-β expression, showing that the change of Th17/Treg cell ratio in sevoflurane anesthetized mice was much more prominent than that in propofol anesthetized mice. Accumulating evidence has showed that propofol contributes to suppress breast cancer recurrence and metastasis [22]. We found that the sevoflurane anesthetized mice showed lower survival rate compared with the propofol anesthetized mice. These results confirmed that propofol sustained Th17/Treg cell balance by increasing Th17 cell accumulation and decreasing Treg cells to promote Th17/Treg ratio, thus attenuating the inhibition of surgical trauma on anti-tumor immune effects, decreasing the risk of postoperative cancer metastasis, and increasing postoperative survival rate of elderly lung cancer patients.

To summarize, this study elaborated that propofol maintained the balance of Th17/Treg cell in elderly patients undergoing lung cancer surgery through GABAA receptor and inhibited metastasis of lung cancer. The correlation between anesthesia and cancer prognosis has aroused the interest of tumor surgery. However, this study has several limitations. (1) We only assessed the effect of propofol on postoperative cancer metastasis and living conditions of aged mice with surgery in animal experiments, whereas perioperative and postoperative prognostic indicators were not evaluated. (2) The function of propofol on other adaptive immunity has been extensively discussed. Compared with sevoflurane, propofol can better facilitate the differentiation of Th cells to Th1 cells and inhibit surgical stress [50]. Joint anesthesia of propofol-sevoflurane is beneficial to the recovery of T/B cell subset activity, attenuation of immunosuppression and inhibition of ALL progression [51]. This study only explored the effect of CD4^+^ cell subsets Th17/Treg ratio and propofol anesthesia on Th17/Treg balance, while their effect on the imbalance of other CD4^+^ cell subsets (such as TH1 and TH2) and other immune cell types (such as CD8^+^) was not investigated. (3) Single propofol treatment condition was set in animal experiment. Effect time- and dose-dependent study was not performed. (4) Clinical sample size was small. Only 36 patients were enrolled in this study due to limited conditions. (5) The tendency of Th17/Treg ratio changes in lung cancer and completely resected lung cancer remains unclear. A previous study found increased Treg cells abd Foxp3 expression, decreased Th17 cells and RORγt expression, diminished Th17/Treg ratio and negative correlation with cancer stages in elderly lung cancer patients [13]. Duan MC et al. selected NSCLC patients aged 37–68 years as subjects and found increased Th17/Treg ratio and corresponding cellular factors relative to healthy people [15], which was opposite to the findings of Zhao L et al. After comparing the differences of these studies, age might exert certain effects on Th17/Treg in lung cancer patients. The immune regulatory mechanism varies in young patients and elderly patients. This study only focused on the effect and mechanism of propofol on Th17/Treg cell balance in elderly lung cancer patients after radical resection of pulmonary carcinoma, which might differ in young patients. Still, further investigations are required for verification. In future studies, we will conduct multi-centered study and enroll bigger sample size to further verify the effect and mechanism of propofol in Th17/Treg cell balance in senile patients with lung cancer during perioperative period. Additionally, more studies are needed to better understand the effect and regulatory mechanism of propofol anesthesia in immune dysfunction in patients with lung cancer after surgery. The existing laboratory studies and clinical trials of propofol are not consistent. Most studies emphasize the effect of propofol on the behavior of cancer cells, but did not explore the underlying mechanism. In the future, we shall carry out more prospective trials to refine our clinical guidance.

## Supplementary Information

**Additional file 1: Figure S1**. Sorting of CD4^+^ T cells in CD4^+^CD25^+^Foxp3^+^Treg cells and CD3^+^CD8^−^ cells in CD3^+^CD8^−^IL-17^+^Th17 cells analyzed by flow cytometry. Fig. S1A is related to Fig. 1A; Fig. S1B is related to Fig. 2C; Fig. S1C is related to Fig. 4A.

**Additional file 1: Figure S2**. Sorting of CD4^+^ T cells in CD4^+^CD25^+^Foxp3^+^Treg cells and CD3^+^CD8^−^ cells in CD3^+^CD8^−^IL-17^+^Th17 cells analyzed by flow cytometry. Fig. S2 is related to Fig. 5A.

**Additional file 1: Figure S3**. Propofol inhibited invasion and migration of lung cancer cells. **A** Effect of different doses (0.3 μg/mL, 3 μg/mL, 30 μg/mL) of propofol on lung cancer cell viability was measured by CCK-8 assay; **B** Cell invasion were detected using Transwell assay; **C** Cell migration was detected Transwell assay; **D** Positive rate of E-cadherin protein was detected by immunocytochemistry and the stronger red fluorescence meant the higher positive rate. All experiments were repeated three times. Data were analyzed using one-way ANOVA, followed by Tukey's multiple comparisons test, **p < 0.01, ***p < 0.001.

## Data Availability

The data that support the findings of this study are available from the corresponding author upon reasonable request.

## Abbreviations

CD4^+^ T: Cluster of differentiation 4^+^ T
GABAA: γ-Aminobutyric acid A
TCI: Target-controlled infusion
PBMCs: Peripheral blood mononuclear cells
qRT-PCR: Quantitative real-time polymerase chain reaction
GAPDH: Glyceraldehyde-3-phosphate dehydrogenase
IgG: Immunoglobulin G
ANOVA: One-way analysis of variance
PMA: Phorbol 12-myristate 13-acetate
PBS: Phosphate buffer saline
IL: Interleukin
HE: Hematoxylin & eosin
FBS: Fetal bovine serum
DMEM: Dulbecco’s modified Eagle’s medium
IL: Interleukin
ELISA: Enzyme-linked immunosorbent assay
CCK-8: Cell counting kit-8
DAB: Diaminobenzidine
RORγt: Retinoid-related orphan receptor γt
Foxp3: Forkhead box P3
