# Supplementary figures and images for: Propofol maintains Th17/Treg cell balance in elderly patients undergoing lung cancer surgery through GABAA receptor
Source: BMC Immunol. 2022 Nov 25;23:58. doi: 10.1186/s12865-022-00490-8 (PMC9701037; doi:10.1186/s12865-022-00490-8)

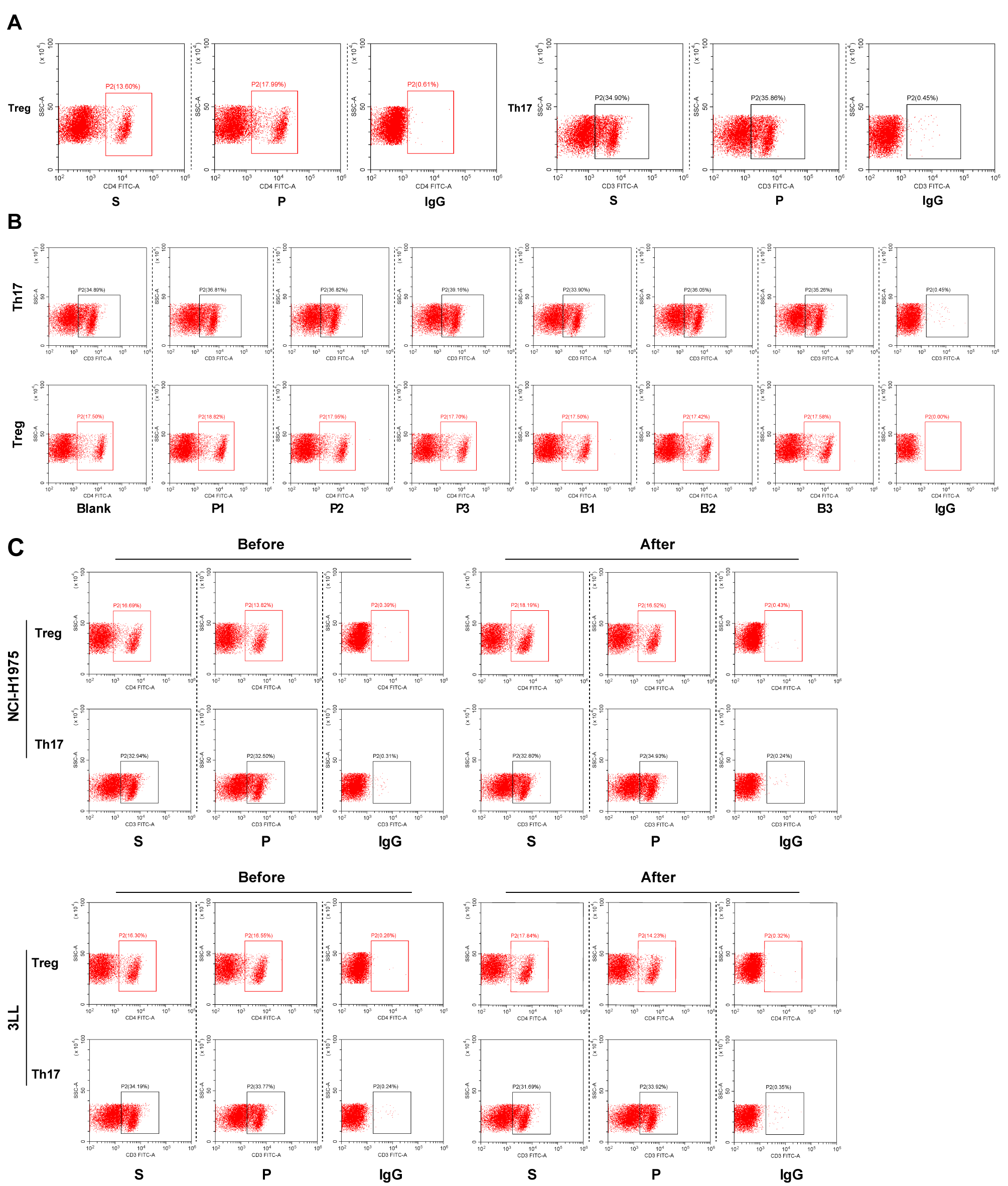

Supplement: Supplementary file 1 — Additional file 1: Figure S1. Sorting of CD4+ T cells in CD4+CD25+Foxp3+Treg cells and CD3+CD8− cells in CD3+CD8−IL-17+Th17 cells analyzed by flow cytometry. Fig. S1A is related to Fig. 1A; Fig. S1B is related to Fig. 2C; Fig. S1C is related to Fig. 4A. [file 12865_2022_490_MOESM1_ESM.tiff]

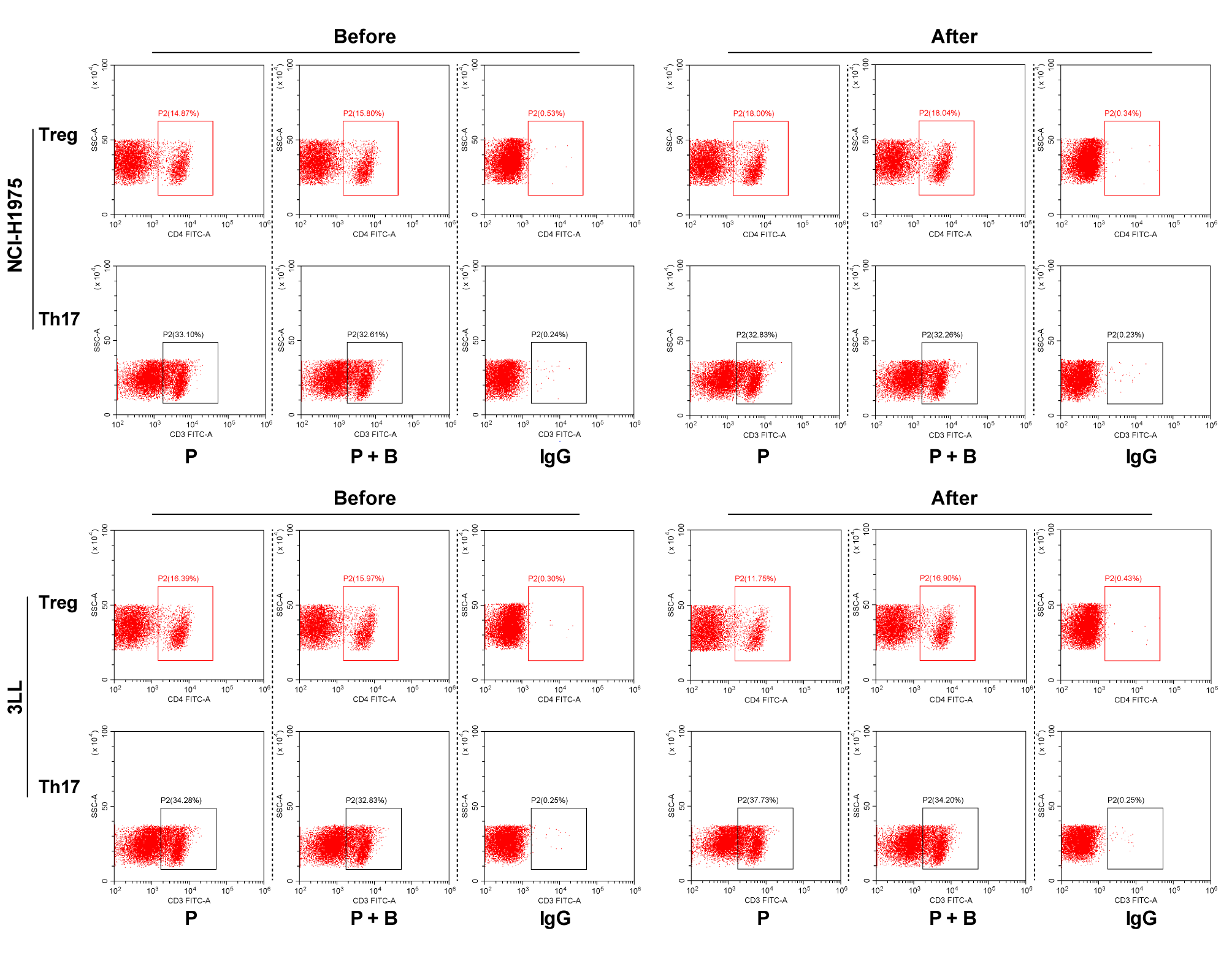

Supplement: Supplementary file 2 — Additional file 1: Figure S2. Sorting of CD4+ T cells in CD4+CD25+Foxp3+Treg cells and CD3+CD8− cells in CD3+CD8−IL-17+Th17 cells analyzed by flow cytometry. Fig. S2 is related to Fig. 5A. [file 12865_2022_490_MOESM2_ESM.tiff]

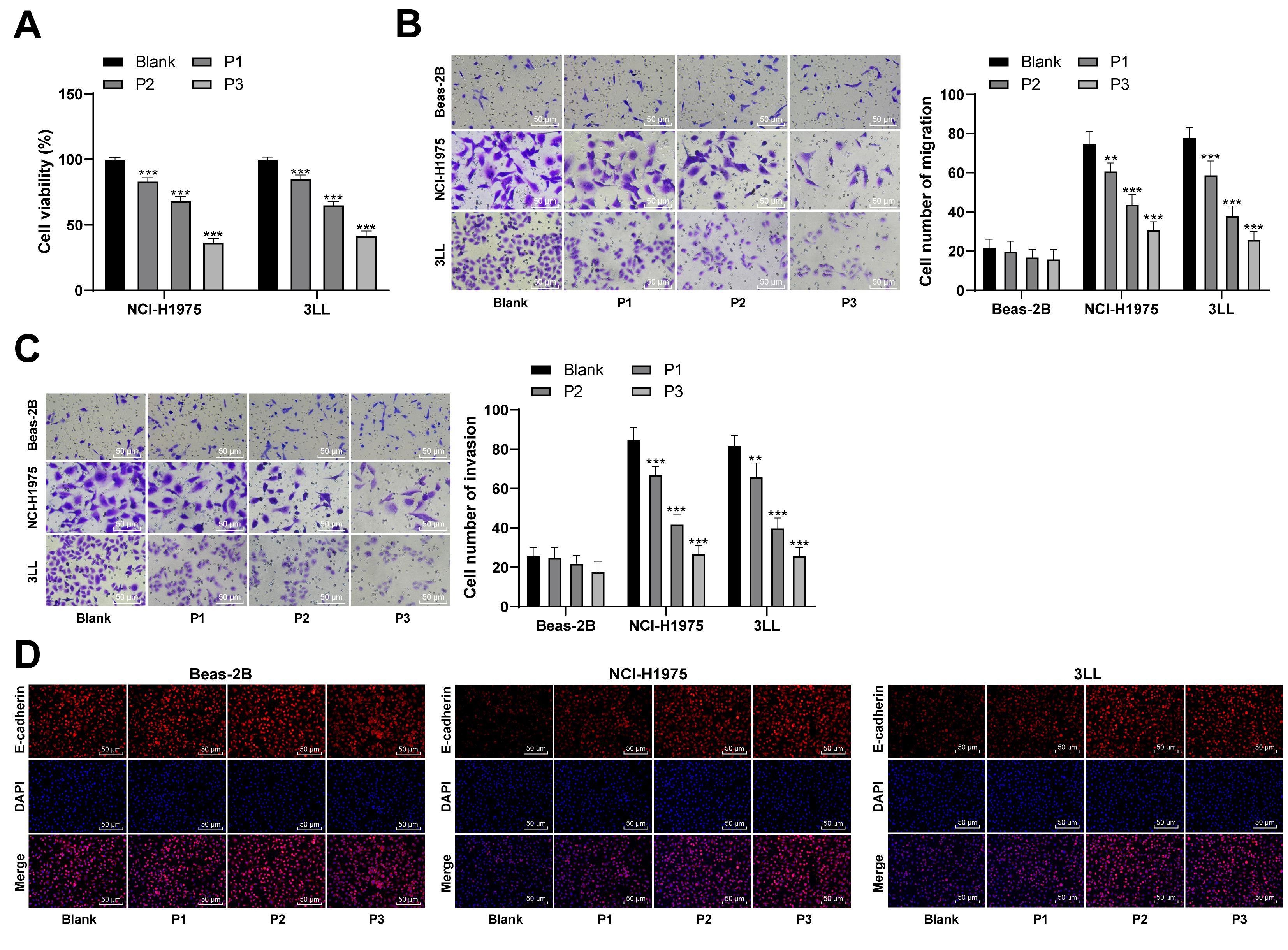

Supplement: Supplementary file 3 — Additional file 1: Figure S3. Propofol inhibited invasion and migration of lung cancer cells. A Effect of different doses (0.3 μg/mL, 3 μg/mL, 30 μg/mL) of propofol on lung cancer cell viability was measured by CCK-8 assay; B Cell invasion were detected using Transwell assay; C Cell migration was detected Transwell assay; D Positive rate of E-cadherin protein was detected by immunocytochemistry and the stronger red fluorescence meant the higher positive rate. All experiments were repeated three times. Data were analyzed using one-way ANOVA, followed by Tukey's multiple comparisons test, **p < 0.01, ***p < 0.001. [file 12865_2022_490_MOESM3_ESM.tiff]
